# *Neisseria meningitidis* carriage rate, antibiotic susceptibility profile, and associated factors among prisoners at Jimma zonal correction facility in Jimma Town, Southwestern Ethiopia: a cross-sectional study

**DOI:** 10.1186/s41182-022-00462-z

**Published:** 2022-09-16

**Authors:** Samuel Assefa, Alemseged Abdissa, Yared Alemu, Lencho Girma, Degemu Sahlu

**Affiliations:** 1 Mizan Aman Health Science College, Mizan Aman, Ethiopia; 2 Armeur Hansen Research Institute, Addis Ababa, Ethiopia; 3 College of Health Science Department of Medical Laboratory, Jimma University, Jimma, Ethiopia; 4 College of Health Science Department of Public Health, Salale University, Fiche, Ethiopia

**Keywords:** *Meningitides*, Serogroup, Prisoner, Pharyngeal, Carriage, Antibiotic, Susceptibility

## Abstract

**Background:**

*Neisseria meningitidis* causes severe life-threatening meningococcal disease with a case fatality rate of 10–15% even with proper treatment. In Ethiopia, particularly in our study area, inadequate information is found on meningococcal disease. So, this study aimed to assess *N. meningitidis* carriage rate, antibiotic susceptibility profile, and associated factors among prisoners in Jimma Town, Southwestern Ethiopia.

**Methods:**

A cross-sectional study was conducted in Jimma town, Southwest Ethiopia, from May to October 2019. A stratified sampling technique was used and proportional allocation was done. A total of 550 oropharyngeal swabs were collected, processed, isolated, and identified *N. meningitidis* using standard microbiological techniques. Antibiotics susceptibility test was done for isolates using the disk diffusion method. Data on demographic and associated factors for carriage were collected using a structured questionnaire. Data were summarized using frequency, percentage, graph, and table. A logistic regression model was used to see the association between the dependent and independent variables. Variables with a *p*-value < 0.25 during bivariate analysis were included in multivariate analysis to identify factors significantly associated with the meningococcal carriage and, a *p*-value < 0.05 was considered statistically significant.

**Result:**

Out of the 550 study participants, 76(13.8%) with (CI: 7.20–18.20) were found carriers of *N meningitidis*. The predominant isolates were non-serogroupable 26(34.2%) and serogroup W/Y 22(28.9%), respectively. *N. meningitidis* isolates showed highest sensitivity to chloramphenicol 74(97.4%). Meningococcal carriage rate was significantly associated with being age group of 16–20 years; having respiratory symptoms within 3 months and active cigarette smoking within 3 months*.*

**Conclusions:**

The majority of participants harbor most of the serogroups responsible for invasive cases of meningococcal disease. Respiratory symptoms, active cigarette smoking, and age group of 16–20 years increased the risk of *N. meningitidis* pharyngeal carriage rate. This study suggests providing better health education to control respiratory symptoms, smoking, and providing antibiotic prophylaxis for prisoners.

## Background

*Neisseria meningitidis* is a Gram-negative, oxidase-positive, aerobic diplococcus known to cause meningitis. There are 13 serogroups of *N. meningitis* that have been identified, of which 6 (A, B, C, W135, Y, and X) are responsible for epidemics and almost all invasive cases of meningococcal diseases like severe sepsis, meningitis, and pneumonia [1, 2]. Humans are the only natural hosts for *N. meningitidis*, which normally colonizes the mucosa of the upper respiratory tract without causing invasive disease, sometimes; infection can spread through the bloodstream to the brain. It is believed that 10% to 20% of the population carries *N. meningitidis* in their throat at any given time. Different factors can enhance carriage rate: immunological susceptibility, poor living condition, overcrowding, housing condition with no or poor ventilation, coincident respiratory tract infections of viral or bacterial origin; active as well as passive smoking; and low socioeconomic status [3]. The bacterium is particularly sensitive to desiccation and transmission from one individual to another requires the acquisition of *N. meningitidis* mainly through direct contact and airborne droplet inhalation [4, 5]

The balance between the carriage of the organism and the development of the disease after the acquisition is affected by *N. meningitidis* characteristics such as bacterial virulence factors, host factors including age, damage of the mucosal barrier, and host immune defense mechanisms and environmental factors [1]. Early antibiotic treatment of meningococcal disease is crucial for keeping the case fatality rate and risk of sequelae as low as possible [6]. Because *N. meningitidis* can be highly contagious, close contacts of the infected patient are treated prophylactically with rifampin, fluoroquinolone, or ceftriaxone [7]

Every year, it is estimated that at least 1.2 million people become sick with meningococcal disease (MD) and 135,000 individuals die worldwide [8]. Meningococcal disease occurs worldwide with incidence rates varying from 1 to 1000 cases per 100,000**.** Meningococcal disease is fatal in as many as 50–80% of untreated cases, and case fatality rates even in treated individuals are about 10–15%. In addition, MD causes great morbidity, with 12–20% of survivors suffering significant permanent clinical sequels (e.g., paralysis, deafness, mental impairment, amputations, and seizures), and learning disabilities. However, MD is often considered endemic globally, although epidemics occur frequently in the meningitis belt in sub-Saharan Africa [8–10]**.** The overall incidence of meningococcal disease in Europe and North America is 1–3/100 000 of the population, but incidence rates may reach 1000/100 000 or 1% of the population during severe epidemics in sub-Saharan Africa [11, 12]. Asymptomatic pharyngeal carriers of *N. meningitidis* account for 5–10% of the general population [13]. The prevalence of meningococcal carriage differs within and between countries, varying across age groups and over time. The rate is also influenced by contact with cases and the epidemic/endemic situation [14]. In Ethiopia, a major epidemic was recorded in 2001 with 6964 cases and 330 deaths. Another epidemic was also recorded in 2003–2004 with 332 cases and 160 deaths [15].

Ethiopia lies in the African Meningitis Belt, and outbreaks of meningitis are reported approximately every 8–12 years. Major outbreaks were recorded in Ethiopia in 1981 (50,000 cases and 990 deaths) and in 1989 (45,806 cases and 1,686 deaths). A risk assessment carried out early 2012 showed that 5 of the regions were at high risk, the remaining 6 regions were at moderate and low risk for meningitis outbreak. Accordingly, the country planned to introduce mass campaign for individuals between 1 and 29 years of age (70% of the total population) over 3 years period in three different phases from 2013 to 2015. The main objective of Men “A” campaign was set to eliminate epidemics of meningococcal meningitis caused by serotype “A” from Ethiopia, thereby to reduce morbidity and mortality among the population by achieving ≥ 95% coverage in all target areas. The total vaccination coverage among the target population in year 2013 and 2014, respectively, were 98.4% and 97.6% [16].

Limited studies identify high-risk populations such as military recruits, prisoners, university students, students living in halls of residence, school children, and those in close contact with cases. Similarly, data regarding pharyngeal meningococcal carriage, serotypes involved, and susceptibility testing for antibiotics conventionally used for treatment and prophylaxis of invasive meningococcal diseases are few in Ethiopia. Therefore, this study aims to determine the characteristics of pharyngeal *N. meningitidis* carriage in one of the high-risk groups of prisoners as well as susceptibility to antimicrobials commonly used for treatment and prophylaxis among asymptomatic carriers of Ethiopian prisoners in Jimma Town.

## Methods

### Study setting, design, and period

A cross-sectional study was conducted among volunteer prisoners from May to October 2019 in Prison located in Jimma town, Jimma zone, Oromia region, Southwest Ethiopia. It is 346 km far from Addis Ababa, the capital city of Ethiopia. There were a total of 2164 inmates; 2054 (94.9%) were male and 110 (5.1%) were female inmates. The prisons average room size was 141 m2, with the smallest room measuring 30 m2 (323 ft2) and the largest one at 195 m2 (2098 ft2) (1517 ft2) (Jimma Zonal Prison daily record and attendance loglogbook019).

### Sample size and sampling technique

**Fig. 1:**
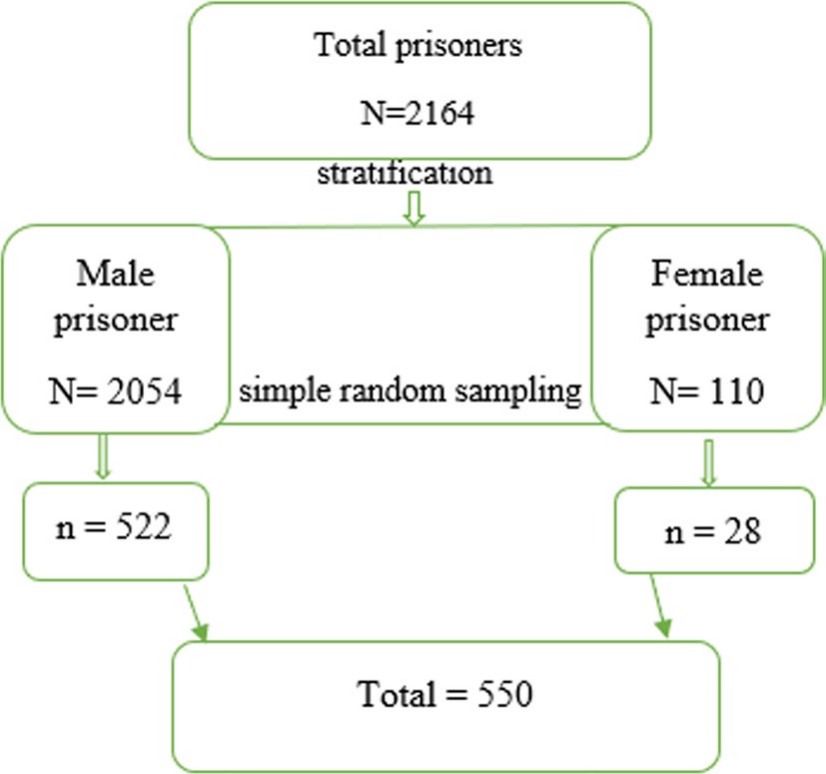
Sampling procedure of study participant to assess Neisseria meningitidis carriage rate, antibiotic susceptibility profile, and associated factors among prisoners at Jimma Town, Southwestern Ethiopia in 2019

The sample size was calculated using the single proportional formula [n = (Zα/2)2 p (1 − p)/d2] by considering the prevalence of 20.4% [17], with a 95% confidence interval, and a 5% margin of error with a 10% non-response rate and design effect of 2. Based on the above assumption, the sample size required was 550. A stratified random sampling technique was employed by forming two strata: (1) strata males consisting of 2054 males and (2) strata females consisting of 110 females. Then the sample size was determined for each stratum by proportional allocation. Finally, simple random sampling was employed by the lottery method to recruit from a prepared list of sample frames in each stratum (Fig. 1).

### Study variables

The dependent variable in this study is Neisseria meningitis carriage while the independent variables are sociodemographic factors; age, sex, educational status, geographic area, crowding/number of inmates per room: behavioral factors; drug use, handwashing, and duration of incarceration and health-related variables; respiratory symptom and previous antibiotic use.

## Measurement

### Data collection and laboratory methods

Data were collected by interview method using a structured and pre-tested questionnaire. The questionnaire was translated into local languages (Amharic and Afaan Oromo) and retranslated by other translators into English. The training was given for the data collectors and the data collected were checked for completeness by principal investigators and double-entry check-in EpiData was done.

The questionnaire contains sociodemographic and associated risk factors, some variables were defined as follows to get accurate data.

Antibiotic use: The participants took antibiotics for fever and diarrhea, for various types of respiratory symptoms such as coughs and colds during the 3 months before 14 days before the pharyngeal swab.

Respiratory symptom: Participants who show common symptoms in the upper and lower respiratory tract include the cold, laryngitis, pharyngitis/tonsillitis, acute rhinitis, acute rhinosinusitis, and pneumonia (CDC, WHO).

#### Prisoner

Any individual involuntarily confined or detained in a penal institution. Individuals incarcerated in a penal institution, and individuals dare detained pending arraignment, trial, or sentencing [18].

#### Passive smoking

Is the inhalation of smoke, called secondhand smoke (SHS), or environmental tobacco smoke (ETS), by persons other than the intended "active" smoker. It occurs when tobacco smoke enters an environment, causing its inhalation by people within that environment [19] and the practice of this must be within the last 3 months of the participant.

#### Active smoking

Inhalation of tobacco smoke directly by participant currently or recently within the last 3 months once per a day.

### Laboratory method

#### Specimen collection and processing

After a written informed consent was obtained, specimens were collected from recruited participants from the posterior wall of the pharynx. Swabs were collected by a trained medical microbiologist using pre-moistened sterile cotton swabs through the open mouth, following standard operating procedure by touching the tip of a swab against the pharyngeal and tonsillar fossa and then passing it in an upward semi-circular motion over the soft palate to the opposite side. After collection, samples were inserted immediately into the Amie charcoal transport medium and transported to the laboratory within one hour in a cold box [20, 21].

#### Laboratory assay for isolation and identification

**Fig. 2:**
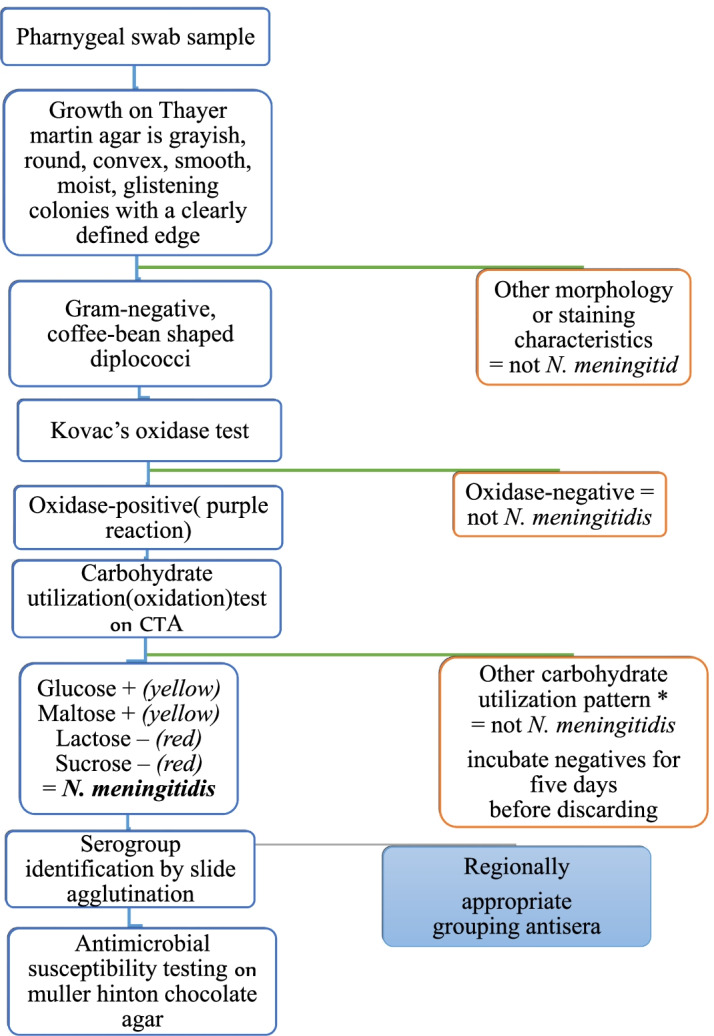
Microbiological standard procedures flowchart, Jimma Ethiopia, 2019

The swabs were plated out immediately after arrival at the laboratory with a maximum delay of 30 min. Isolation and identification was carried out in the microbiology laboratories of Jimma University, Jimma, Ethiopia. Pharyngeal swabs were plated onto selective modified Thayer–Martin (MTM) agar by rolling and streaking swabs over plates using a sterile loop. MTM was supplemented with vancomycin, colistin, nystatin, and trimethoprim (VCNT) inhibitor. Inoculated plates were incubated at 35–37˚C under a 5–10% CO2-enriched atmosphere for 72 h. Plates were examined for the growth of colonies every 24 h. The grown colonies on the plates were first tested for Gram reaction and those giving colonies, which demonstrated Gram-negative diplococci results were further tested for oxidase activity. Then, all oxidase-positive, Gram-negative diplococci were sub-cultured on chocolate agar before performing biochemical tests to ensure adequate viability. Carbohydrate utilization tests (glucose, maltose, lactose, and sucrose) on cystine trypticase agar (CTA) were used to further confirm colonies. Gram-negative, oxidase-positives, maltose positive, lactose negative, and sucrose negative diplococci were counted as N meningitidis. Once identified as N, meningitidis serogroup of colonies was done by slide-agglutination method using A, B, C, and W /Y standard antisera to identify A, B, C, and W/Y serogroups of *N. meningitidis*, respectively. Serogroups A, B, C, and W /Y were prioritized as the likely most prevalent serogroups. The presence of agglutination during mixing is used to check for the specific serotypes (Fig. 2). Colonies that showed no agglutination or agglutinate for more than one antisera were classified as non-serogroupable (NG) meningococci [22]. All prepared media were tested for growth support, production of proper biochemical reactions, and susceptibility using the ATCC quality strain of (*N. meningitis* serogroup A (ATCC) received from the Ethiopian public health institute. The plates were checked for sterility before use by incubating at 35–37˚C and 5–10% CO2-enriched atmosphere. Serogrouping of isolates was done after mixing colonies with normal saline to check for auto-agglutination. Standard antisera were also mixed with manufacturer positive polyvalent control and negative controls separately on a slide to confirm purity. Finally, standard antisera test for serogroup A (ATCC) strain [22].

#### Antibiotic susceptibility testing

Susceptibility testing for antibiotics used for the treatment of patients for prophylaxis (prophylactic treatment) was done using the disc diffusion method. A standardized 0.5 McFarland was used to confirm inoculum density. Colonies were inoculated on Muller Hinton chocolate agars prepared from sheep blood and inoculated at 37 °C under a 5–10% CO2-enriched atmosphere for 24 h thereafter at 24-h intervals until 96 h. Susceptibility to antibiotics was confirmed according to the Clinical and Laboratory Standard Institute (CLSI-2017) of anti-microbial chemotherapy guidelines for penicillin, ceftriaxone, chloramphenicol, ciprofloxacin, and rifampicin and determined for all isolates, using (Liofilchem, Oxoid) anti-microbial product [23].

#### Data management and analysis

The collected data were entered by using Epi-Data version 4.6 and exported to SPSS 23 software for cleaning, recording, categorizing, and analyzing. Bivariate analysis was done to see the association between independent and outcome variables. Those variables with a *P*-value < 0.25 during the bivariate analysis were included in the multiple logistic regression analysis to assess the relative effect of confounding variables. Since the outcome variable is categorical type, the adjusted odds ratio was calculated by multiple logistic regressions model. The model fitness was checked by using Hosmer and Lemeshow’s goodness of fit test and the model was fit since. It had *p*-value greater than 0.05. After multivariate analysis had been done, adjusted odds ratio (OR) was used to measure the strength of association between the dependent variable and the independent variable, while 95% CI and *P* value be used to assess whether the association is significant.

## Results

### Sociodemographic, behavioral, and health-related factor

There were a total of 550 participants were included with 100% response rate in this study, of which 526 (95.64%) were males and 24 (4.36%) were females. The participants mean ± SD age of was 28 ± 3.21 years; 33.82% of them were between the ages of 16 and 20; 25.82% were between the ages of 21 and 25; and 23.28% were older than the age of 30. The majority of the participants (55.63%) were rural residents and 47.99% attended primary school, while 27.27% had no formal education. 15.73% of participants were active smokers, 28.7% were passive smokers, 15.3% chewed khat, and 43.6% had not used drugs in the past three months, while 39.6% reported having respiratory symptoms and 42.9% reported having taken antibiotics for fever and diarrhea as well as for different respiratory symptoms such as coughs and colds (Table 1).

**Table 1:** Sociodemographic, behavioral and health-related characteristics of prisoners at correction facility in Jimma Town, Southwestern Ethiopia in 2019

Factors	Non-carrier	Carrier
	*n* = 474 (86.2%)	*n* = 76 (13.8%)
Age category		
16–20 years	142 (25.82%)	44 (8%)
21–25 years	120 (21.82%)	22 (4%)
26–30 years	90 (16.36%)	4 (0.72%)
Older than 30 years	122 (22.18%)	6 (1.10%)
Sex		
Male	452 (82.20%)	74 (13.45%)
Female	22 (4%)	2 (0.36%)
Residential background		
Rural	260 (47.27%)	46(8.36%)
Urban	214 (38.91%)	30 (5.45%)
Educational status		
No education	132 (24%)	18 (3.27%)
Primary	228 (41.45%)	36 (6.54%)
Secondary	98 (17.82%)	20 (3.64%)
Diploma and above	16 (2.91%)	2 (0.36%)
Active smoking		
Yes	46 (8.36%)	40 (7.37%)
No	428 (77.82%)	36 (6.54%)
Passive smoking		
Yes	136 (24.73%)	22 (4%)
No	338 (61.45%)	54 (9.82%)
Chewing Khat		
Yes	68 (12.36%)	16 (2.92%)
No	406 (73.82%)	60 (10.93%)
Nondrug user		
Yes	228 (41.45%)	12 (2.18%)
No	246 (44.73%)	64 (11.63%)
Incarceration history		
Yes	22 (4%)	6 (1.1%)
No	452 (82.18%)	70 (12.73%)
Hand washing habit		
With water alone	352 (64%)	62 (11.22%)
With soap and water	122 (22.18%)	14 (2.54%)
Respiratory symptom		
Yes	174 (31.64%)	44 (8%)
No	300 (54.54%)	32(5.82%)
Antibiotic use		
Yes	216 (39.27%)	20 (3.64%)
No	258 (46.91%)	56 (10.18%)

### Serogroups of *N. meningitidis and* distribution by age group

**Fig. 3:**
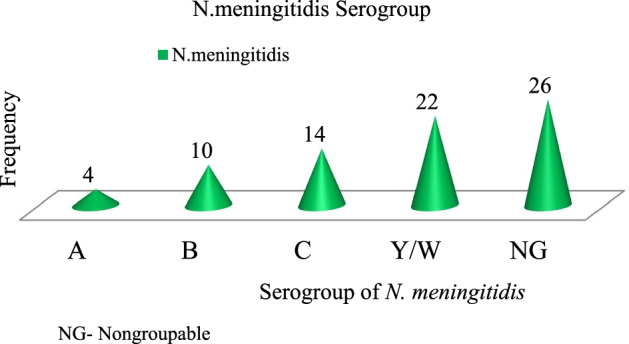
Characteristic of isolates serogroup, Jimma Ethiopia, 2019

Out of the 550 study participants, 76(13.8%) with (CI: 7.20–18.20) were found carriers of N meningitidis. In this study, 76 *N. meningitidis* isolates were recovered, of which 26 (34.2%) were non-groupable (NG) meningococci. The most abundant serogroup out of 50 (65.8%) serogroupable *N. meningitidis* isolates was W/Y (28.9%), followed by serogroups C, B, and A, which accounted for 18.4%, 13.2%, and 5.3% of all isolates, respectively (Fig. 3).

The serogroups distribution across different age groups showed that, in the 16–20 and above 30 age groups only serogroups A and B were found. Serogroup C was only found in 16–20 and 21–25 age groups, while serogroup W/Y was recovered from all age groups except the above 30 age group participants. The majority (57.9%) of the isolates were recovered from individuals aged 16 to 20, while 28.9%, 7.9% and 5.3% of the isolates were recovered from participants aged 21 to 25, above 30 and 26 to 30, respectively. The 16–20 age group had a higher prevalence of serogroups B, C, and W/Y than the other age groups (Table 2).

**Table 2:** Distribution of serogroups of *Neisseria meningitidis* isolate by age group among prisoners at correction facility in Jimma Town, Southwestern Ethiopia, 2019

	Age group				Total
	16–20 years	21–25 years	26–30 years	Above 30 years	
A	2 (2.6%)	0	0	2 (2.6%)	4 (5.3%)
B	8 (10.5%)	0	0	2 (2.6%)	10 (13.2%)
C	10 (13.2%)	4 (5.3%)	0	0	14 (18.4%)
W/y	12 (15.8%)	8 (10.5%)	2 (2.6%)	0	22 (28.9%)
NG	12 (15.8%)	10 (13.2%)	2 (2.6%)	2 (2.6%)	26 (34.2%)
Total	44 (57.9%)	22 (28.9%)	4 (5.3%)	6 (7.9%)	76 (100.0%)

*NG* non-groupable

### Antimicrobial susceptibility pattern of *N. meningitis* isolates

*N. meningitidis* isolates showed the highest sensitivity to chloramphenicol 74(97.4%), followed by rifampicin 72(94.7%), ceftriaxone 64(84.2%), ciprofloxacin 60(78.9%) and to penicillin 48(63.2%). Isolates showed no resistance to rifampicin and exhibited relatively high resistance to ceftriaxone (15.8%), ciprofloxacin 6 (7.9%), and penicillin 4 (5.3%). Meanwhile, 24(31.6%), 10(13.2%) and 4(5.3%) of the isolates showed intermediate resistance to penicillin, ciprofloxacin and rifampicin, respectively (Table 3).

**Table 3:** Antibiotic susceptibility profile of *Neisseria meningitidis* isolated from prisoners at correction facility in Jimma Town, Southwestern Ethiopia, 2019

Antibiotics	Antibiotic susceptibility			Total
	Sensitive	Intermediate	Resistance	
Penicillin	48 (63.2%)	24 (31.6%)	4 (5.3%)	76 (100%)
Chloramphenicol	74 (97.4%)	0	2 (2.6%)	76 (100%)
Ceftriaxone	64 (84.2%)	–	12 (15.8%)	76 (100%)
Rifampicin	72 (94.7%)	4 (5.3%)	0	76 (100%)
Ciprofloxacin	60 (78.9%)	10 (13.2%)	6 (7.9%)	76 (100%)

### Factors associated with *N. meningitidis* carriage rate

There were no statistically significant differences in sex, educational status, place of residence, drug use, handwashing, or incarceration history between *N. meningitidis* carriers and non-carriers. Multivariate logistic regression results revealed that factors that were independently associated with the *N. meningitidis* carriage rate were age, respiratory symptoms, antibiotic use, and active cigarette smoking. Being in the 16–20 age group was about five times more likely to become a carrier of *N. meningitidis* than being older than 30 years old, and active smokers were 6.8 times more likely to become a carrier of *N. meningitidis* than non-active smokers were. Antibiotic users, on the other hand, were less likely to become *N. meningitis* carriers than non-users (Table 4).

**Table 4:** Bivariate and multivariate logistic regression analysis to assess factors associated with *Neisseria meningitidis* carriage rate among prisoners at correction facility in Jimma Town, Southwestern Ethiopia, 2019

Variables	Carrier	No carrier	COR (95% C. I)	p-value	AOR (95% C. I)	p-value
	No. (%)	No. (%)				
Active smoking						
Yes	40 (46.5)	46 (53.5)	10.34 (6.00, 17.80)	0.000	6.788 (3.007, 15.326)	0.000
No	36 (7.8)	428(92.2)	1			
Passive smoking						
Yes	22 (13.9)	136 (86.1)	1.01 (0.59, 1.73)	0.963		
No	54 (13.8)	338 (86.2)	1			
Chewing khat						
Yes	16 (19.0)	68 (81.0)	1.592 (0.86, 2.92)	0.331		
No	60 (12.9)	406 (87.1)	1			
Drug user						
Yes	12 (5.0)	228 (95.0)	0.202 (0.10,0.38)	0.000	0.112 (0.10,1.38)	0.079
No	64 (20.6)	246 (79.4)	1			
Age category						
16–20 years	44 (57.9)	142 (30.0)	6.30 (2.59, 15.29)	0.000	5.310 (1.404,20.076)	0.014
21–25 years	22(28.9)	120 (25.3)	3.73 (1.46, 9.51)	0.004	3.171 (.782, 12.865)	0.106
26–30 years	4 (5.3)	90 (19.0)	0.90 (0.24, 3.29)	0.878	0.866 (.130, 5.754)	0.882
> 30 years	6 (7.9)	122(25.7)	1		1	
Sex						
Male	74 (97.4%)	452 (85.9%)	0.56 (0.13, 2.41)	0.426	0.48 (0.23, 2.21)	0.321
Female	2 (8.3%)	22 (14.1%)	1			
Residence						
Rural	46 (60.5)	260 (54.9)	1.26 (0.76, 2.06)	0.355		
Urban	30 (39.5)	214 (45.1)	1			
Educational level						
No education	18 (23.7)	132 (27.8)	1.09 (0.23, 5.14)	0.219	4.210 (0.404,16.076)	0.341
Primary school	36 (47.4)	228 (48.1)	1.26 (0.27, 5.72)	0.760	2.171 (0.782, 10.865)	0.098
Secondary school	20 (26.3)	98(20.7)	1.63 (0.34, 7.66)	0.23	0 .866 (0.130, 5.754)	0.156
Diploma and above	2 (26.3)	16(3.4)	1		1	
Hand washing habit						
With water alone	62 (15.0)	352 (85.0)	1.53 (0.82, 2.84)	0.169	1.13 (0.82, 2.84)	0.241
With soap and water	14 (10.3)	122 (89.7)	1		1	
Incarceration history						
Yes	6 (21.4)	22 (78.6)	1.76 (0.68, 4.49)	0.23	1.327 (0.007, 3.180)	0.432
No	70 (13.4)	452 (86.6)	1		1	
Respiratory symptom						
Yes	44 (20.2)	174 (79.8)	2.37 (1.44, 3.87)	0.000	2.327 (1.007, 5.380)	0.048
No	32(9.6)	300 (90.4)	1		1	
Antibiotics use						
Yes	20 (8.5)	216 (91.5)	0.42 (0.24, 0.73)	0.001	0.263 (0.106, 0.655)	0.004
No	56 (17.8)	258 (82.2)				

*AOR* adjusted odds ratio, *COR* crude odds ratio, *CI* confidence interval

## Discussion

This study found a 13.8% pharyngeal meningococcal carriage prevalence among asymptomatic prisoners, which is higher than the pharyngeal carriage prevalence of 6.6% reported among the 1–29 age group asymptomatic population in Arba Minch southern part of Ethiopia [5]. Similarly, a lower prevalence of pharyngeal *N. meningitidis* carriers was reported in Mali (5.0%) among the general population [24] and 9% in Brazil, among 1–24 age group population [25]. This difference may be due to the study setting.

Historically, semi-closed populations had high rates of meningococcal carriage and experienced recurrent outbreaks like university students and military camps [26]. While studies conducted among college freshmen in North India [27] and students aged 18–24 in Chile [28] found carriage rates of 1.5% and 4%, respectively, which were lower than in this study. The difference may be due to the difference in the participants (prisoners vs. students), the difference in setting (prison vs. school), and the country’s economic state. The meningococcal carriage rate in closed populations like professional soldiers serving in Poland in 2016 indicated by 5.2% carriage rates [29]. All of the above studies had lower carriage rates of *N. meningitidis* than in this study. This may be due to the different socioeconomic and political differences among the countries.

However, higher pharyngeal meningococcal carriage was reported; 20.4% *N. meningitis* nasopharyngeal carriage from Addis Ababa, Ethiopia, among apparently healthy school children and adolescents [17] and an overall 15% *N. meningitidis* carriage rate was reported from the USA among university students from 2015–2016 [30] compared to 13.8%. Similar to this study *N. meningitidis* carriage rate was reported in Portugal among undergraduate university students in 2014, indicated by a pharyngeal meningococcal carriage rate of 13.3% [31]. Among closed populations, lower carriage rate than in this study was reported from Koreas in 2012 among university freshmen students who were admitted to a dormitory, indicated by 12.9% of *N. meningitidis* overall carriage rates [32]. The difference may be due to the difference in the participants (prisoners vs. students), difference in setting (prison vs school), and the country’s economic and political level difference.

In multivariable analysis being 16–20 years of age, active smoking and having respiratory symptoms in the past 3 months were significantly associated with an increased carriage rate of *N. meningitidis*. Being 16–20 years of age and smoking were positively associated with *N. meningitidis* carriage rate and also recently exposed to antibiotic use was negatively associated with *N. meningitidis* carriage rate. Being 16–20 years of age was about five times more risk for *N. meningitidis* carriage rate as compared with older than 30 years prisoners and active smokers were 6.8 times more likely to develop *N. meningitidis* than not active smokers. On the other hand, antibiotic users were less likely to develop *N. meningitis* compared with non-user. The above finding was comparable with studies documented in the United Kingdom and US America. Age groups from 15–19 years showed a significant association with increased *N. meningitidis* carriage rate documented in Brazil was comparable with this study [25]. The evidence of active smoking associated with increasing of meningococcal carriage rate in the Poland military is also comparable with this study [29]. Similar to this study, respiratory tract infections and active smoking were high risks of becoming meningococcal carriage in Korea [32].

*N. meningitidis* is divided into 13 serogroups based on the immunological specificity of the capsular polysaccharide. It has been known that pathogenic strains are encapsulated and six of these serogroups (A, B, C, W135, Y, and X) cause more than 90% of the invasive disease worldwide [32]. This study identified serogroup A 4(5.3%), serogroup B 10(13.2%), serogroup C 14(18.4%), serogroup W/Y 22(28.9%), and 26(34.2%) non-serogroup able variant with low carriage prevalence of serogroup A observed. Our study almost identified similar serogroup distribution with serogroup documented among apparently healthy school children and adolescents in Addis Ababa, Ethiopia, and among the general normal population at Arba Minch, Ethiopia, where serogroups W135, C, A, B, X, Y, and NG variants were identified. However, at Arba Minch, Ethiopia, no serogroup A carriage was found. The introduction of the monovalent serogroup A conjugate vaccine is thus expected to influence by lowering the carriage prevalence of serogroup A [5, 17]. A study conducted in Jimma, southwest Ethiopia, identified *N. meningitidis* serogroup ACYW, which was similar to this study except for serogroup B.

Comparing the meningococcal carriage serogroup of this study to studies of other regions found some similarities and variations in serogroup distribution. For example, serogroups were documented in Brazil (C, B, and NG) (36); in Chile (B, W, and NG) [28]; in Koreas (C, B. NG 29E, and W13) [32]; in United States (B, C, W, X, Y, NG) [30]; in Poland (A, B, E29, C, Y, and W) [29]; in Mali (W Y and NG) [24] and United Kingdom( B Y, C, X, and W) [33] were obvious for similarities and variations in serogroups distribution with this study. The variation may be because of the setting and period difference.

In this study isolates of *N. meningitis* exhibited sensitivity to a penicillin (63.2%): exhibited sensitivity to ceftriaxone (84.2%) and ciprofloxacin (78.9%). However, the isolates were highly susceptible to chloramphenicol (97.4%) and rifampicin (94.7%). Reduced penicillin susceptibility was documented in Swedish 2% isolates were resistant to penicillin [7]; in Malaysian, 12.5% resistant to penicillin [34] and in 18 African countries within the meningitis belt (2%) reduced susceptibility to penicillin [35] were less than reduced susceptibility isolates of *N. meningitis* exhibited in this study. However, in the study reported in Addis Ababa, Ethiopia, only 4.5% sensitivity to penicillin was higher than the reduced susceptibility exhibited in isolates of *N. meningitis* in this study [17]. In contrast, in the study reported in Jimma, Ethiopia, isolates were susceptible to penicillin [36]. Reduced susceptibility of *N. meningitis* isolates to ciprofloxacin was comparable with a study in Addis Ababa, Ethiopia, of sensitivity to ciprofloxacin (83.7%) [17]. However, the resistance of *N. meningitis* isolates (60%) to ciprofloxacin in Gurage Zone, Ethiopia, was higher than the resistance to ciprofloxacin exhibited in this study [37]. Isolates of *N. meningitis* 84.2% sensitivity to ceftriaxone in this study was comparable with the study reported (87%) sensitive to ceftriaxone in Gurage Zone, Ethiopia [37]. The resistance documented in Addis Ababa, Ethiopia (69.4%), to ceftriaxone was higher than ceftriaxone resistance in this study [17]. The high susceptibility of rifampin (94.7%) and chloramphenicol (97.4) in isolates of *N. meningitis* in this study was comparable with studies that reported (7%) resistance to rifampin and (4%) resistant to chloramphenicol in Gurage Zone, Ethiopia, respectively [37]. Resistance to chloramphenicol (30%) in Gondar, Ethiopia, was higher than the resistance to chloramphenicol in this study [37].

## Limitation

In this study, serogroup X was not tested because it was impossible to avail serogroup antiserum during the study period. However, serogroup X was an emerging serogroup that was responsible for the epidemic in the African meningitis belt in the recent decade.

## Conclusions

Generally, in this study one-eighth of the study participants were carriers of *N. meningitidis*. The participants harbor most of the serogroups responsible for invasive cases of meningococcal disease and the predominantly capsulated isolates were serogroup W/Y. The low carriage rate of serogroup A was isolated which was previously responsible for the occurrence of the epidemic in the meningitis belt. The isolates exhibited resistance to ceftriaxone, penicillin, and ciprofloxacin. Respiratory symptoms, active cigarette smoking, and age group of 16–20 years increased the risk of *N. meningitidis* pharyngeal carriage rate.

## Recommendation

This study suggests the government provide for all prisoner’s drug prophylaxis preventive measures to prevent the circulation of *N. meningitidis* serogroup in the setting of heavily overcrowded living conditions to prevent and control respiratory symptom in further. This study suggests the Jimma zonal administration commission control smoking by health education and applying rule and regulation in the prison.

## Data Availability

The data sets used and/or analyzed during the current study are available from the corresponding author on reasonable request.
